# Identification of potential core genes in colorectal carcinoma and key genes in colorectal cancer liver metastasis using bioinformatics analysis

**DOI:** 10.1038/s41598-021-03395-5

**Published:** 2021-12-14

**Authors:** Lipeng Niu, Ce Gao, Yang Li

**Affiliations:** 1 Graduate School, Hebei North University, Zhangjiakou, 075000 Hebei China; 2 Fuyong People’s Hospital, Shenzhen, Guangdong 518103 China; 3 Shihua Residential District Community Health Service Center, 12th Xiangzhou Road, Jinshan District, Shanghai, 201500 Shanghai China

**Keywords:** Biotechnology, Cancer

## Abstract

Colorectal carcinoma (CRC) is one of the most prevalent malignant tumors worldwide. Meanwhile, the majority of CRC related deaths results from liver metastasis. Gene expression profile of CRC patients with liver Metastasis was identified using 4 datasets. The data was analyzed using GEO2R tool. GO and KEGG pathway analysis were performed. PPI network of the DEGs between 1 and 2 gene sets was also constructed. The set 1 is named between primary CRC tissues and metastatic CRC tissues. The set 2 is named between primary CRC tissues and normal tissues. Finally, the prognostic value of hub genes was also analyzed. 35 DEGs (set 1) and 142 DEGs (set 2) were identified between CRC liver metastatic cancer patients. The PPI network was constructed using the top 10 set 1 hub genes which included AHSG, SERPINC1, FGA, F2, CP, ITIH2, APOA2, HPX, PLG, HRG and set 2 hub genes which included TIMP1, CXCL1, COL1A2, MMP1, AURKA, UBE2C, CXCL12, TOP2A, ALDH1A1 and PRKACB. Therefore, ITIH2 might represent the potential core gene for colon cancer liver metastasis. COL1A2 behaves as a key gene in colorectal carcinoma.

## Introduction

Colorectal cancer (CRC) is the third most prevalent cancer in the world, both in males and females. With an estimated 147,950 new cases and 53,200 deaths in 2020, it is the second most common cause of cancer-related death in the USA^1^. The liver is the most common site for metastatic CRC. Particularly, 15–25% of CRC patients present with liver metastases at the time of diagnosis^2^, which is the main cause of CRC related deaths^3^. Identifying mechanism underlying liver metastasis in CRC patients can help uncover biomarkers for early diagnosis and development of corresponding targeted chemotherapy^4^.

In this study, core genes associated with CRC liver metastases were identified using bioinformatic analyses. We first identified differentially expressed genes (DEGs) between primary CRC and metastatic CRC tissues using data in the Gene Expression Omnibus (GEO) database. Gene Ontology (GO) and Kyoto Encyclopedia of Genes and Genomes (KEGG) enrichment analysis were then performed to identify biological processes and pathways regulated by the DEGs. PPI network of the top 10 most regulated hub genes related to liver metastasis in CRC patients was then constructed using STRING package. The prognostic utility of the hub genes in liver metastatic CRC patients was performed using GEPIA platform.

## Materials and methods

### Data source

We obtained gene expression profile data from the GEO database based on the keywords “colorectal carcinoma”, “liver metastasis”, and “homo sapiens”. From the search results, select a dataset that has both primary CRC and liver metastatic CRC tissues and normal colon tissue. At last, we filter out 4 more suitable datasets (GSE6988, GSE41258, GSE49355 and GSE81558). Data for expression profiles of 45,195 genes in CRC patients with liver metastasis in four datasets was downloaded from GEO database (https://www.ncbi.nlm.nih.gov/geo/).

### Identification of DEGs

DEGs between primary CRC and metastatic CRC tissues (set 1) were identified using the GEO2R online tool (https://www.ncbi.nlm.nih.gov/geo/geo2r/) as previously described^5^. DEGs between primary CRC tissues and normal colon tissue (set 2) were identified using the same method. Duplicate and invalid genes were removed using excel. Significant gene expression was based on statistical significance at P < 0.05 and |logFC|> 1. The data was analyzed using the Venn diagram webtool (bioinformatics.psb.ugent.be/webtools/Venn/). Venn diagram of DEGs was then generated in SVG format.

### Pathways and biological processes regulated by the DEGs

Biological process (BP), molecular function (MF) and cellular component (CC) regulated by the DEGs were identified using GO analysis. KEGG was also performed to identify biological pathways regulated by the DEGs^6^. Both analyses were performed using an online tool (DAVID), available at https://david.ncifcrf.gov/. Statistical significance was set at P < 0.05 and gene counts > 5.

### Identification of hub genes and construction of the PPI network

Interacting DEGs in metastatic CRC patients were identified using the Search Tool for the Retrieval of Interacting Genes (STRING) (http://string-db.org) as previously described^7^. The PPI network was then constructed and visualized using Cytoscape software. The protein nodes were calculated using a cytoscape plugin and cytoHubba. The top 10 most dysregulated genes were selected as the hub genes.

### Kaplan–Meier survival analyses of the hub genes

The gene expression profiling in cancerous and normal colon tissues was performed using GEPIA^8^. Survival analysis of metastasis CRC patients was based Kaplan–Meier Survival analyses, using GEPIA (http://gepia.cancer-pku.cn/) tool. Based on the expression of each hub gene, the cancer patients were divided into low or high expression group based on the median mRNA expression of hub genes, at statistical significance of P < 0.05.).

### Image analyses for human samples from The Human Protein Atlas

Pathological section images of human patient CRC tumor tissues were obtained from The Human Protein Atlas (www.proteinatlas.org).We obtained expression of potential genes in CRC from the Human Protein Atlas based on the keywords “ITIH2”and COL1A2.

### Ethical statement

Our study did not require an ethical board approval because it did not contain human or animal trials.

## Results

### Identification of DEGs

Herein, we assessed gene expression profiles in four datasets; GSE6988, GSE41258, GSE49355 and GSE81558. GSE6988 contained data for 52 primary tumor tissues, 28 metastasis tumor tissues and 28 normal tissues. GSE41258 contained gene expression data for 186 primary tumor tissues, 47 metastasis tumor tissues and 54 normal tissues. GSE49355 contained data for 20 primary tumor tissues, 19 metastasis tumor tissues and 18 normal tissues. GSE81558 contained data for 23 primary tumor tissues, 19 metastasis tumor tissues and 9 normal tissues. The data is summarized in Table 1.

**Table 1:** Gene expression data extracted from the four GEO datasets.

Dataset	Primary	Metastasis	Normal	Total number
GSE6988	52	28	28	108
GSE41258	186	47	54	287
GSE49355	20	19	18	57
GSE81558	23	19	9	51

Significant differential gene expression was based on P < 0.05 and |logFC|> 1. Overall, we identified 6065 dysregulated genes, in which 2324 were upregulated, whereas 3741 were down regulated. The upregulated/down-regulated genes were 309/417 (726) in GSE6988, 427/681 (1108) in GSE41258; 681/1503 in GSE49355 (2184) and 907/1140 (2047) in GSE81558 dataset.

Two more sets of DEGs were identified. Set 1 comprised DEGs between metastatic and primary tumor tissues, whereas set 2 included DEGs between primary tumor and normal colon tissues.

**Figure 1:**
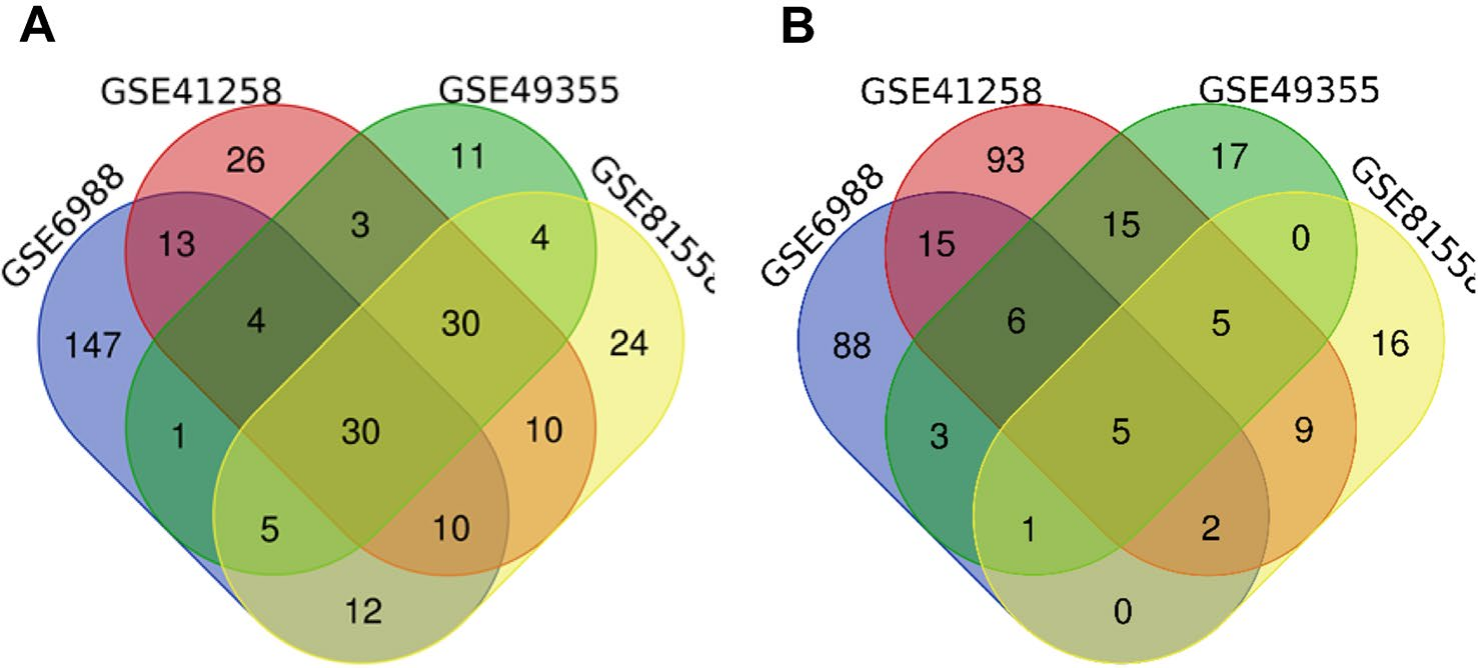
(SET 1) Venn diagram showed 35 DEGs distinguishing primary tumor tissues and metastasis tumor tissues in both GEO datasets. (**A**) 30 Upregulated genes (**B**). 5 Downregulated genes. *DEG* differentially expressed gene, *GEO* Gene Expression Omnibus.

**Figure 2:**
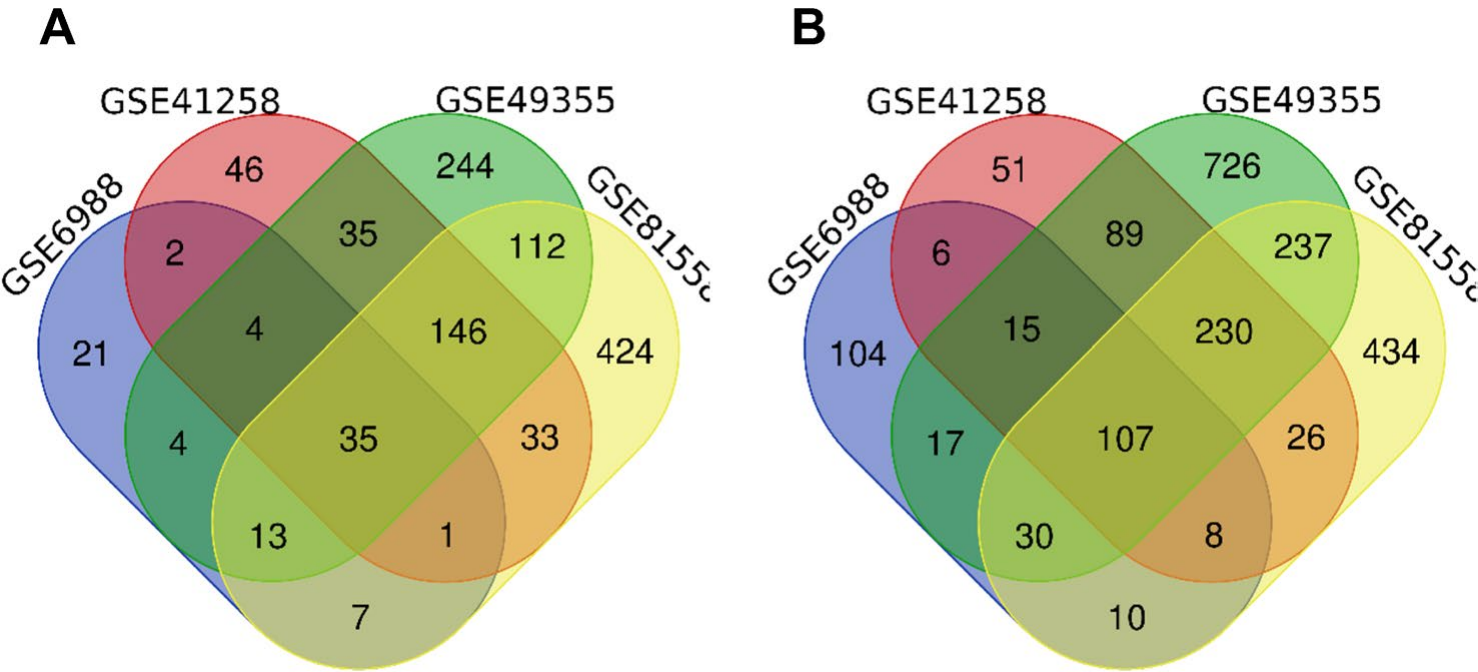
(SET 2) Venn diagram showed 142 DEGs distinguishing primary tumor tissues and normal colon tissues in both GEO datasets. (**A**) 35 Upregulated genes (**B**). 107 Downregulated genes. *DEG* differentially expressed gene, *GEO* Gene Expression Omnibus.

Venn analysis was performed to identify the intersecting set 1 and set 2 DEGs (Fig. 1). Also, there were 35 DEGs among the four groups. For set 1, there were 30 upregulated and 5 downregulated genes (35). In set 2, there were 142 DEGs, in which 35 were upregulated whereas 107 were downregulated (Fig. 2).

### Functional enrichment analyses

GO analysis revealed that set 1 DEGs mainly regulated BPs, including *platelet degranulation, acute-phase response* and *negative regulation of endopeptidase activity* (Table 2). Further CC analysis revealed that set 2 DEGs regulated *blood microparticle, extracellular region, extracellular space, extracellular exosome and platelet alpha granule lumen*. MF analysis on its part revealed that set 1 DEGs participated in *serine-type endopeptidase inhibitor and serine-type endopeptidase activities*. KEGG analysis revealed set 1 DEGs mainly regulated *Complement and coagulation cascades*.

**Table 2:** The biological processes and pathways regulated by DEGs (set 1).

Category of gene function	Type of analysis	Gene function	Count	P value
BP	GO:0002576	Platelet degranulation	10	2.15E−13
BP	GO:0006953	Acute-phase response	8	6.66E−13
BP	GO:0010951	Negative regulation of endopeptidase activity	8	2.45E−09
CC	GO:0072562	Blood microparticle	18	3.74E−27
CC	GO:0005576	Extracellular region	26	8.79E−20
CC	GO:0005615	Extracellular space	22	5.25E−16
CC	GO:0070062	Extracellular exosome	26	5.80E−14
CC	GO:0031093	Platelet alpha granule lumen	7	7.19E−10
MF	GO:0004867	Serine-type endopeptidase inhibitor activity	6	8.52E−07
MF	GO:0004252	Serine-type endopeptidase activity	6	9.32E−05
KEGG pathway	hsa04610	Complement and coagulation cascades	6	2.44E−07

*BP* biological process, *CC* cellular component, *DEG* differentially expressed gene, *GO* Gene Ontology, *KEGG* Kyoto Encyclopedia of Genes and Genomes, *MF* molecular function.

GO analysis revealed that set 2 DEGs regulated BP including *regulation of blood pressure* (Table 3). CC further revealed the DEGs participated in *extracellular exosome, extracellular space, proteinaceous extracellular matrix* and so on. Based on MF analysis, the DEGs regulated *metalloendopeptidase activity as well as structural constituent of muscle and calcium ion binding*. KEGG analysis revealed that the DEGs regulated *Mineral absorption*.

**Table 3:** Biological process and pathways regulated by the DEGs (set 2).

Category of gene function	Type of analysis	Gene function	Count	P value
BP	GO:0008217	Regulation of blood pressure	6	1.81E−04
CC	GO:0070062	Extracellular exosome	47	2.04E−07
CC	GO:0005615	Extracellular space	27	1.08E−05
CC	GO:0005578	Proteinaceous extracellular matrix	11	4.29E−05
CC	GO:0030018	Z disc	7	3.01E−04
CC	GO:0005576	Extracellular region	24	0.00268
CC	GO:0005737	Cytoplasm	55	0.00707
CC	GO:0048471	Perinuclear region of cytoplasm	12	0.00849
MF	GO:0008307	Structural constituent of muscle	6	2.11E−05
MF	GO:0004222	Metalloendopeptidase activity	6	0.00221
MF	GO:0005509	Calcium ion binding	14	0.00525
KEGG pathway	hsa04978	Mineral absorption	6	1.61E−04

*BP* biological process, *CC* cellular component, *DEG* differentially expressed gene, *GO* Gene Ontology, *KEGG* Kyoto Encyclopedia of Genes and Genomes, *MF* molecular function.

### Construction of PPI network and identification of hub gene

**Figure 3:**
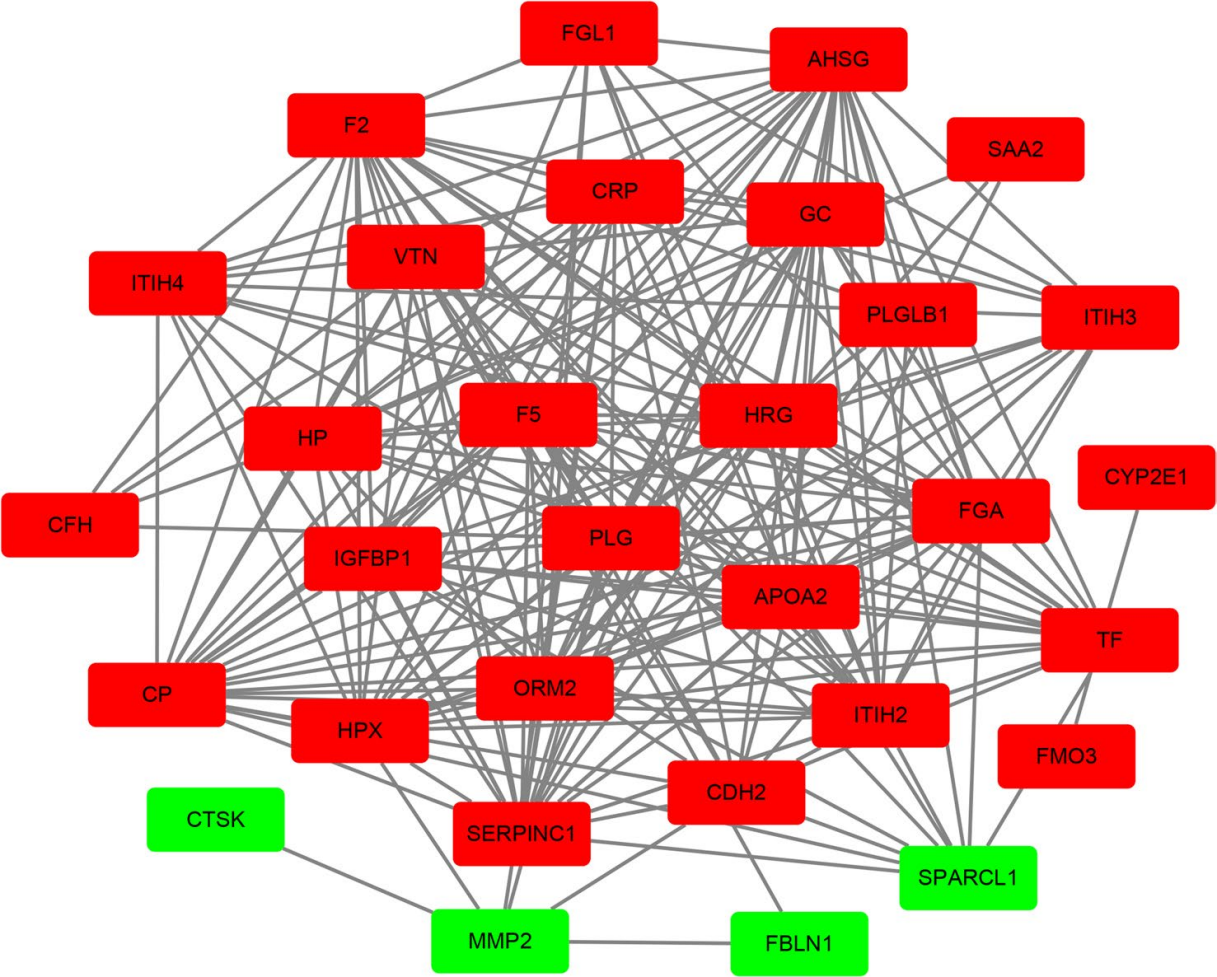
Protein–protein interaction network of proteins encoded by differentially expressed genes between primary tumor tissues and metastasis tumor tissues. Red nodes represent upregulated genes, whereas the green ones represent downregulated genes.

Proteins coded by DEGs were predicted using STRING tools. The PPI network of proteins coded by set 1 DEGs contained 35 nodes and 218 edges (Fig. 3). The top 10 most dysregulated set 1 DEGs were *AHSG, SERPINC1, FGA, F2, CP, ITIH2, APOA2, HPX, PLG* and *HRG* (Table 4).

**Table 4:** Proteins for the 10 most dysregulated DEGs.

Gene symbol	Gene function	Degree
AHSG	α2-Heremans-Schmid glycol	24
SERPINC1	Serpin family C member 1	23
FGA	Fibrinogen alpha chain	22
F2	Coagulation factor II	22
CP	Ceruloplasmin	21
ITIH2	Inter-alpha-trypsin inhibitor heavy chain 2	21
APOA2	Apolipoprotein A	21
HPX	Hemopexin	20
PLG	plasminogen	19
HRG	Histidine rich glycoprotein	18

**Figure 4:**
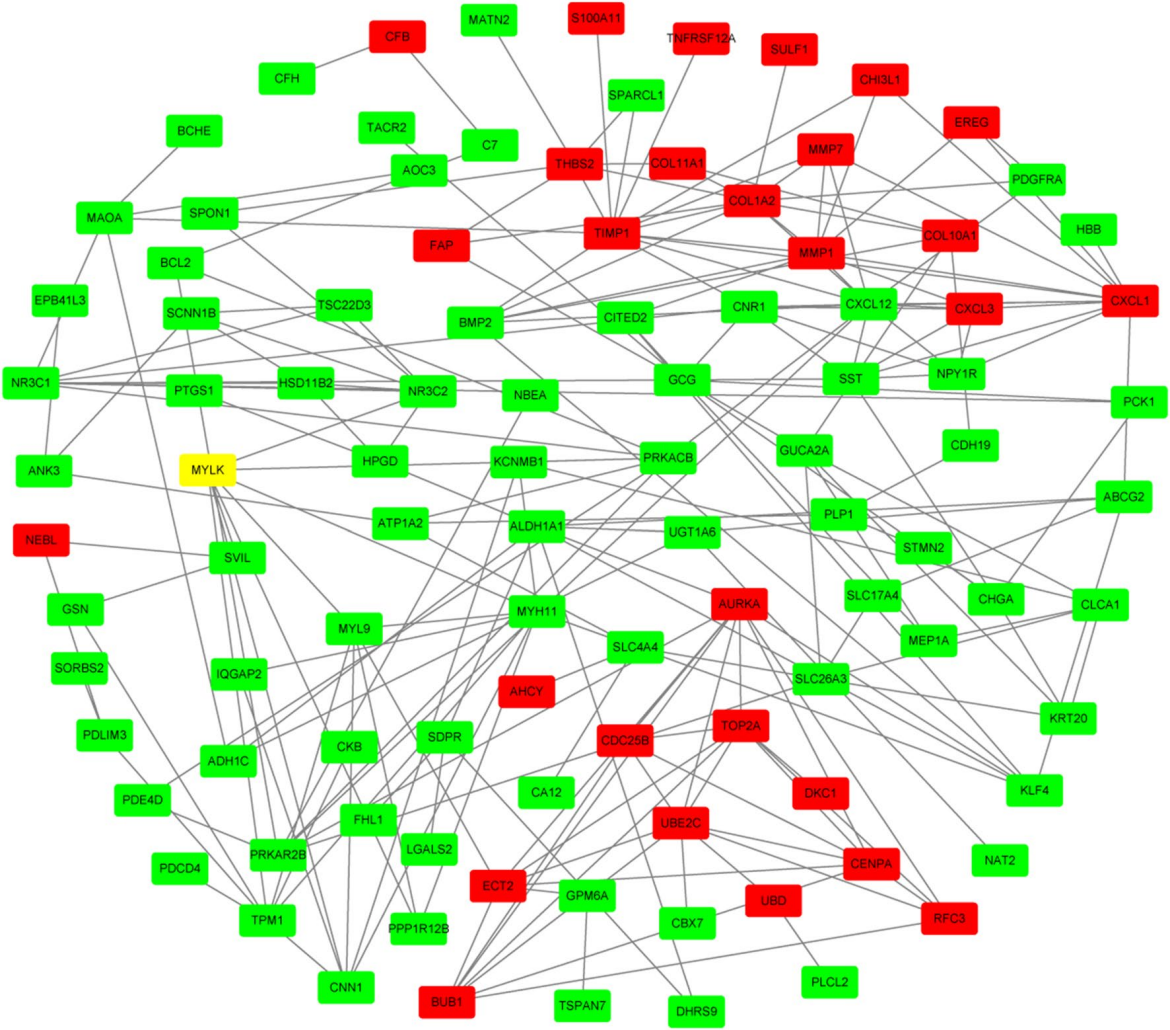
Protein–protein interaction network of proteins encoded by differentially expressed genes between primary tumor tissues and normal colon tissues. Red nodes represent upregulated genes; green nodes represent downregulated genes.

PPI of proteins coded by set two DEGs were constructed using STRING tools. There were 142 nodes and 222 edges in the PPI network of proteins related to set 2 DEGs (Fig. 4). The top ten most dysregulated set 2 DEGs were *TIMP1, CXCL1, COL1A2, MMP1, AURKA, UBE2C, CXCL12, TOP2A, ALDH1A1* and *PRKACB* (Table 5).

**Table 5:** The Top 10 in network string_interactions.tsv ranked by Degree method.

Gene symbol	Gene function	Degree
TIMP1	TIMP metallopeptidase inhibitor 1	13
CXCL12	C-X-C motif chemokine ligand 12	13
CXCL1	C-X-C motif chemokine ligand 1	12
COL1A2	Collagen type I alpha 2 chain	11
AURKA	Aurora kinase A	10
MMP1	Matrix metallopeptidase 1	10
GCG	glucagon	10
UBE2C	Ubiquitin conjugating enzyme E2 C	9
ALDH1A1	Aldehyde dehydrogenase 1 family member A1	8
PRKACB	Protein kinase cAMP-activated catalytic subunit beta	8

### Kaplan–Meier survival analyses of the hub genes

**Figure 5:**
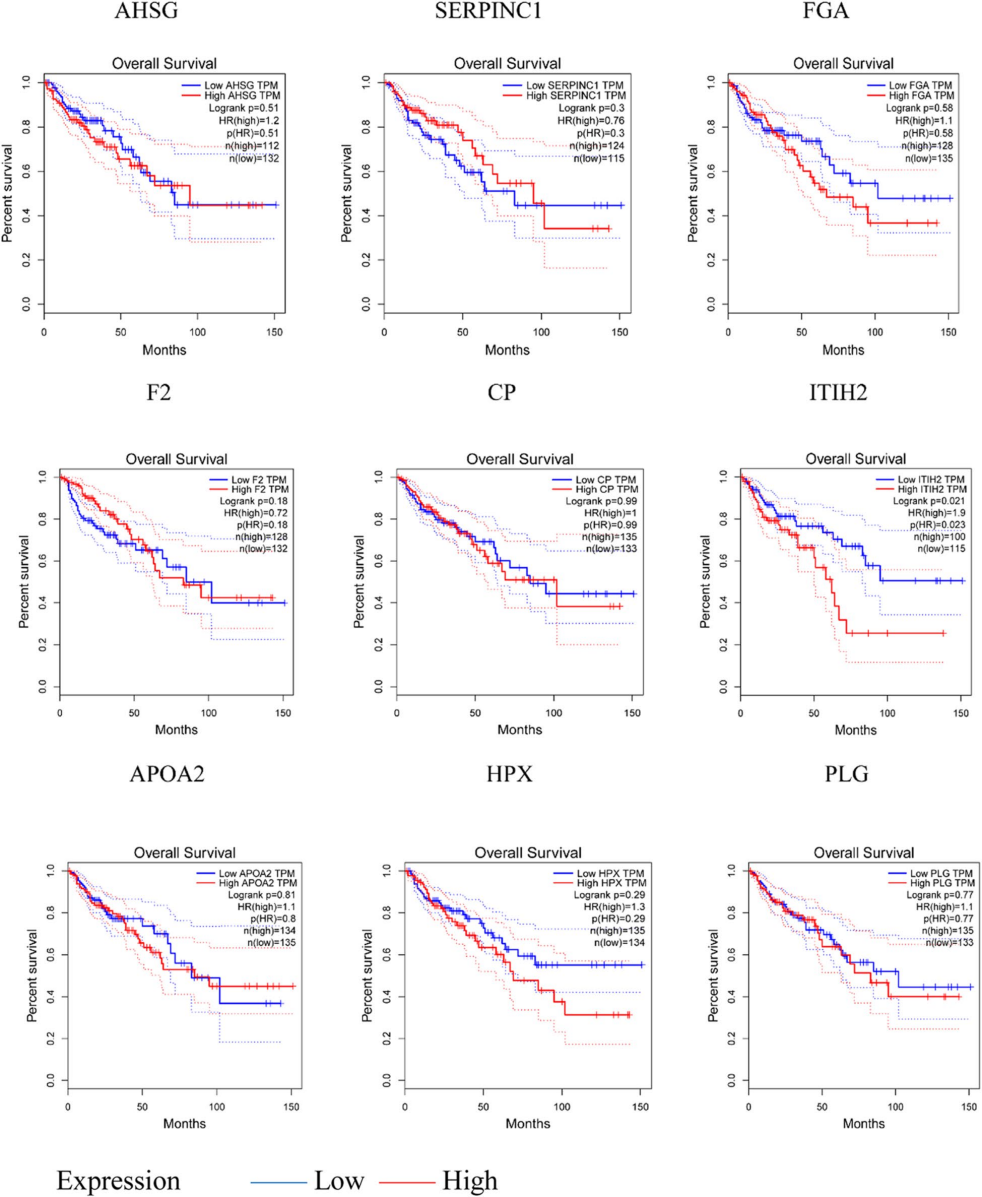
Kaplan–Meier survival analyses of DEGs between primary tumor tissues and metastasis tumor tissues. Over-expression of ITIH2 was associated with poor overall survival. See Table 4 for gene description.

**Figure 6:**
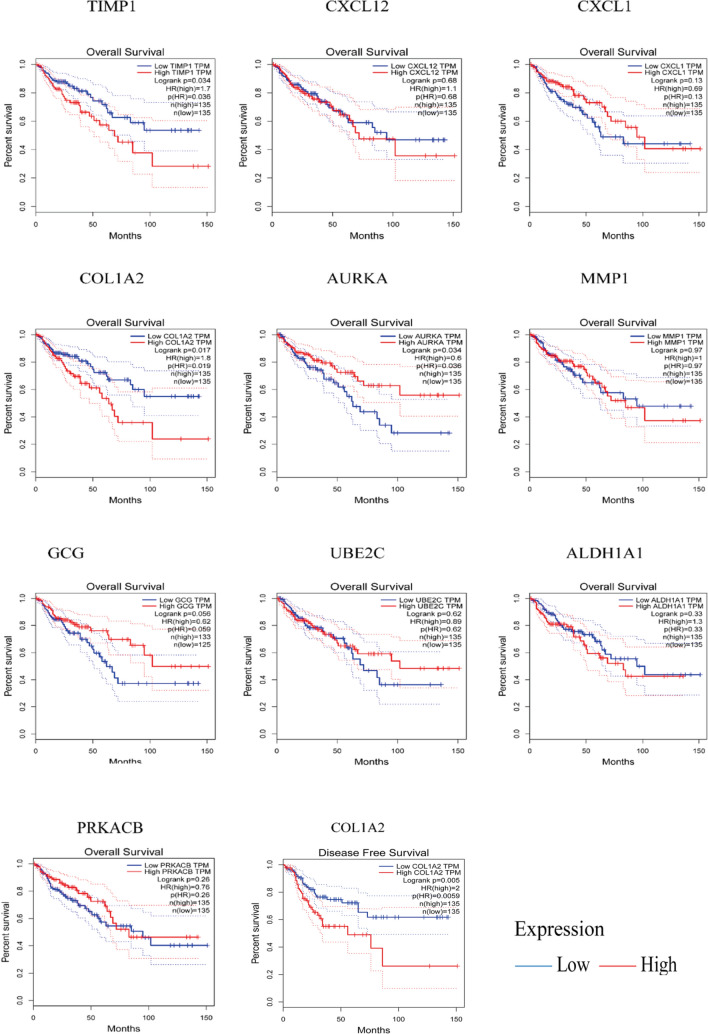
Kaplan–Meier survival analyses of DEGs between primary tumor tissues and normal colon tissues. Under-expression of TIMP1, COL1A2 and AURKA genes, was associated with longer overall survival. Under-expression of COL1A 2 gene was associated with longer disease-free survival of CRC patients. See Table 5 for gene description.

The Kaplan–Meier survival analyses of the top ten set 1 and 2 DEGs in CRC patients with liver metastasis was evaluated using GEPIA tool. Over-expression of the hub genes (ITIH 2, TIMP1, COL1A2 and AURKA) was associated with poor overall survival. Under-expression of ITIH 2 (set one, P = 0.021) was associated with longer overall survival (Fig. 5). Under-expression of TIMP1 (set two, P = 0.034), COL1A2 (set two, P = 0.017) and AURKA genes (set two, P = 0.034), was associated with longer overall survival. Under-expression of COL1A 2 gene (set two, P = 0.017) was associated with longer disease-free survival of CRC patients (Fig. 6).

### Image analyses for human samples from The Human Protein Atlas

ITIH2 and COL1A2 were selected from the website (https://www.proteinatlas.org/).

**Figure 7:**
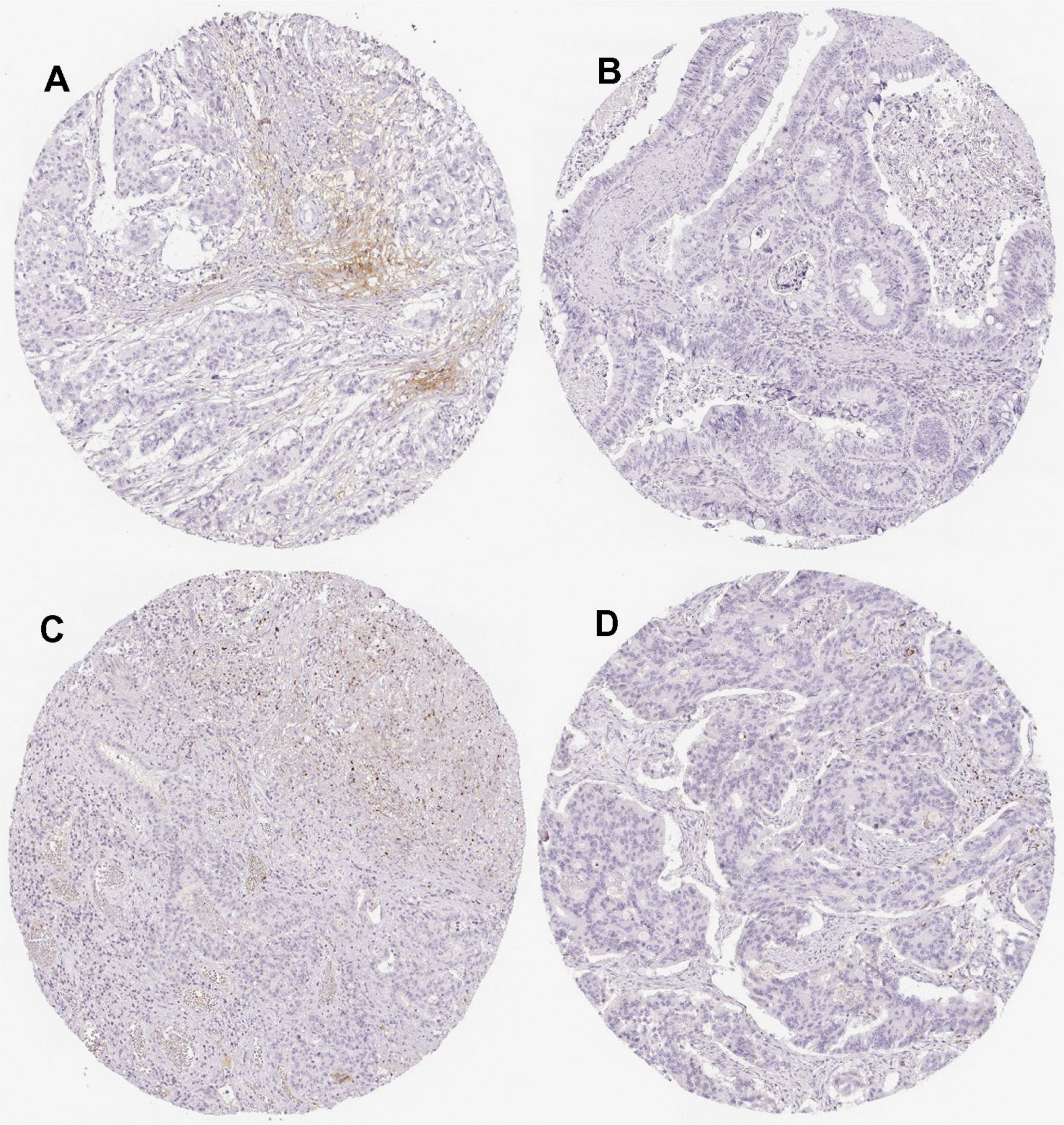
Pathological section images of expression of ITIH2 in CRC were taken from the Human Protein Atlas online database. See Table 6 for gene description.

**Figure 8:**
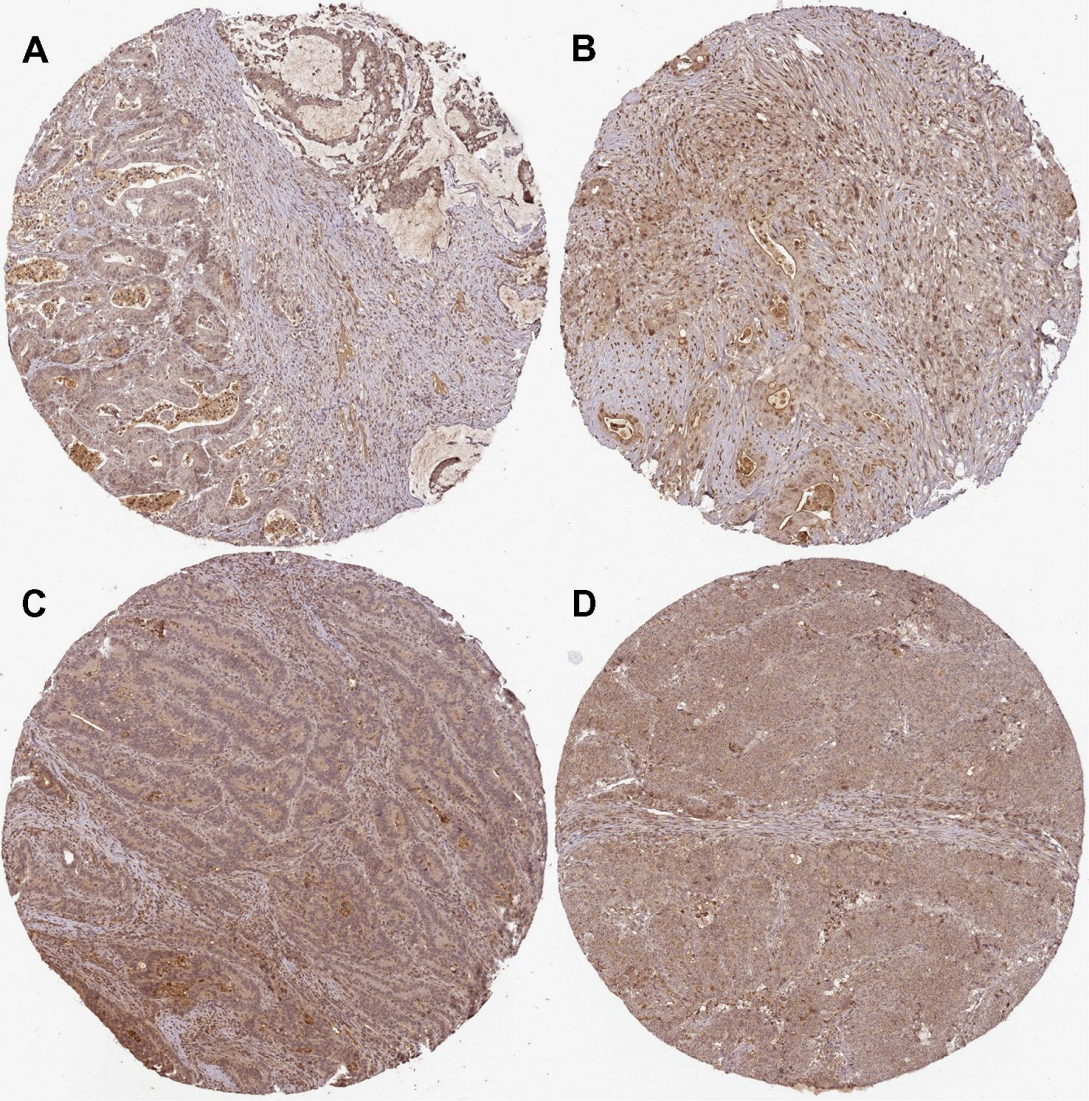
Pathological section images of expression of COL1A2 in CRC were taken from the Human Protein Atlas online database. See Table 7 for gene description.

The pathological section images of the expression of ITIH2 and COL1A2 genes were represented in Figs. 7, 8 and Tables 6, 7).

**Table 6:** Patient of expression of ITIH2 in CRC information sheet.

Figure 7	Gender	Age	Staining	Intensity	Quantity	Location
A	Male	77	Not detected	Negative	None	None
B	Male	65	Not detected	Negative	None	None
C	Female	62	Not detected	Negative	None	None
D	Female	67	Not detected	Negative	None	None

**Table 7:** Patient of expression of COL1A2 in CRC information sheet.

Figure 8	Gender	Age	Staining	Intensity	Quantity	Location
A	Female	84	Medium	Moderate	> 75%	Cytoplasmic/membranous nuclear
B	Female	46	Medium	Moderate	> 75%	Cytoplasmic/membranous nuclear
C	Female	66	Medium	Moderate	> 75%	Nuclear
D	Female	75	Medium	Moderate	> 75%	Cytoplasmic/membranous nuclear

## Discussion

In this study, we identified genes and mechanisms underlying liver metastases in CRC patients. We identified 35 DEGs between metastatic tumor and primary tumor tissues, in which 30 were upregulated whereas 5 were downregulated. Another 142 DEGs between primary tumor and normal colon tissues were also identified. Of these, 35 were upregulated whereas 107 were downregulated.

Set one differently expressed SERPINC1, FGA, F2, HPX and PLG genes regulated the expression of coagulation factor. SERPINC1, also known as ATIII and THPH7, is a member of the serpin C family. SerpinC1 gene encodes antithrombin III (ATIII), a serine protease inhibitor^9^. SERPINC1 inhibits thrombin-induced tumor growth and angiogenesis, impairing proliferation and migration of cancer cells^10^. Fibrinogen alpha chain (FGA) also known as Fib2 participates in hemostasis and wound healing by inducing conversion of fibrinogen to fibrin. FGA protein consists of 2 sub-units, each composed of Aα, Bβ and γ 3 polypeptides encoded by FGA, FGB and FGG genes, respectively^11^. Research shows that CRC cells express FGA. Intriguingly, high levels of phosphorylated FGA have been observed in CRC tissues^12^.

Coagulation factor II, thrombin factor 2(F2) is also known as RPRGL2, THPH1 or PT. Proteolytic cleaving of coagulation factor II generates activated serine protease thrombin. Over-production of thrombin not only increases blood coagulation, but also promotes growth and metastasis of tumors. Accordingly, thrombin and factors contributing to thrombin production are treatment targets for cancer and cancer-associated thrombosis^13^. Hemopexin (HPX), also known as HX encodes a plasma glycoprotein that binds heme with high affinity. HPX also scavenges labile heme. Studies have demonstrated an inverse association between plasma labile heme and Hx in prostate cancer (PCa) patients. Furthermore, under-expression of Hx in PCa tissues correlates with poorly differentiation of the tumor tissues and early relapse^14^. Also, hemopexin promotes invasion of the pancreatic cancer cells. Our findings suggest that hemopexin promotes lymph node metastasis of CRC cells, thus it is a potential therapeutic target for CRC^15^.

PLG encodes a serine protease plasminogen that circulates in blood plasma as an inactive zymogen. It is however converted to the active protease plasmin by several plasminogen activators. Herein, PLG and FGG were over-expressed in NSCLC tissues, relative to paired adjacent normal tissues (P = 0.000). A similar trend was observed in urine of NSCLC patients vs healthy individuals (P = 0.000). PLG and FGG proteins are therefore potential markers for NSCLC diagnosis^16^. Particular, PLG expression is associated with favorable prognostic in patients with advanced grade III/IV FIGO) (advanced ovarian cancer)^17^. Increasing evidence shows that thrombin regulates every step of cancer metastasis: (1) invasion, detachment from primary tumor, migration; (2) entry in to circulation; (3) survival in the vasculature; (4) extravasation and (5) implantation in host organs. Recent molecular data shows that it participates in transendothelial migration, platelet/tumor cell interactions, angiogenesis and other processes. While, thrombin-antithrombin complex (TAT) and vascular endothelial growth factor (VEGF) are over-expressed in CRC patients. Surprisingly, these proteins tend to increase in the course of chemotherapy. SERPINC1, ITIH2, F2 and PLG genes are associated with serine protease^18,19^. Inter-alpha-trypsin inhibitors (ITI) are a family of structurally related plasma serine protease inhibitors that participate in extracellular matrix stabilization and modulation of tumor metastasis. ITIH2 is usually expressed in normal brain tissue and low-grade CNS tumors, but its expression is lost in high-grade CNS tumors including gliobastoma multiforme, further underlining its potential role as an anti-invasive protein^20^.

AHSG, α2-Heremans-Schmid glycol, also referred to as protein Fetuin-A, is almost entirely secreted and expressed in the liver. Expression pattern of AHSG accurately predicts the prognosis of liver cirrhosis and hepatocellular cancer^21^. The positive association between AHSG expression and the risk of developing CRC stems from the effect of AHSG on obesity and insulin resistance^22^. Several studies have demonstrated the strong relationship between insulin resistance as well as hyperinsulinemia and risk of developing CRC^23^. Fetuin‐has been implicated in adhesion of tumor cells, which promotes metastases^24^. Ceruloplasmin (CP) also known as CP-2 is a metalloprotein that binds most of the copper in plasma and participates in peroxidation of Fe (II) transferrin to Fe (III). Given that the expression of Ceruloplasmin is associated with advanced T stage and perineural invasion, it is a potential prognostic marker for bile duct cancer. Under-expression of CP is associated with for poor prognosis of Adrenal cortical carcinoma (ACC). Meanwhile, Ceruloplasmin is a promising (prognostic) marker for pancreatic ductal adenocarcinoma (PDAC) in patients negative for CA19-9^25,26^. Apolipoprotein A (APOA2) also known as apoAII, Apo-AII or ApoA-II, encodes apolipoprotein (apo-) A-II, the second most abundant high density lipoprotein particles. Compared to CA19-9 alone, a combination of CA19-9 and ApoA2-ATQ/AT detects pancreatic cancer up to 18 months earlier than the traditional methods. As such, it can be used for initial pancreatic cancer diagnosis prior imaging^27^. Expression of ApoA1, ApoA2 and ApoA4 is under-expressed, whereas those of tumor antigens (e.g. carcinoembriogenic antigen) and inflammatory markers (e.g., C-reactive protein) are up-regulated in CRC patients, relative to healthy individuals^28^. Histidine rich glycoprotein (HRG) also known as HPRG, HRGP and THPH11, is found in plasma and platelets and contains two cystatin-like domains. Under-expression of HRG is an independent poor prognostic for pancreatic ductal adenocarcinoma (PDAC)^29^. Modulated HRG expression in patients with advanced LC is associated with advanced disease stage and hypofibrinolysis^30^.

In set two DEGs, members of TIMPs family including TIMP1 are natural inhibitors of matrix metalloproteinases (MMPs) including MMP1. TIMP metallopeptidase inhibitor 1(TIMP1) also known as EPA, HCl and CLGI gene, belongs to the TIMP family. Recent evidence demonstrates that even though senescent cells inhibit tumorigenesis in the initial stages of cancer development, they can promote tumor progression in the latter stages. TIMP1 deletion allows senescence cells to promote metastasis, whereas inhibition of senescent cells using a senolytic BCL-2 inhibitor impairs metastasis. Given that TIMP1 promotes tumorigenesis and metastasis of human colon cancer, it is a potential prognostic biomarker for the cancer. Presence of TIMP1 mRNA in platelet independently predicts the presence of colorectal cancer. Transportation of the TIMP1 RNAs to colorectal cancer cells by platelets promote development of colorectal cancer^31,32^. Matrix metallopeptidase 1(MMP1)/CLG/CLGN gene encodes a member of the peptidase M10 family of matrix metalloproteinases (MMPs). Expression of MMP1 in ovarian cancer tissues correlates with poor prognosis. Moreover, EVs with MMP1 mRNA in cancer ascites induces apoptosis of mesothelial cells^33^. MMP-8 and TIMP-1 in serum, but not MMP-9 are associated with poor prognosis of CRC. In CRC patients without systemic inflammation, expression of MMP-8 and TIMP-1 is associated with poor prognosis^34^. TIMP-1 and MMP-7 are highly sensitive and accurate diagnostic biomarkers for metastatic colorectal cancer. The levels of TIMP-1 and MMP-7 levels strongly and positively correlate with the severity and prognosis of liver disease^35^.

CXCL1 and CXCL12 are members of the CXC subfamily of chemokines. C-X-C motif chemokine ligand 1(CXCL1) also known as FSP, GROa or MGSA gene, encodes a member of the CXC subfamily of chemokines. Colorectal carcinoma cells secrete VEGFA, which stimulates tumor-associated macrophages to produce CXCL1 in the primary tumor. High levels of CXCL1 in premetastatic liver tissue recruits CXCR2-positive myeloid-derived suppressor cells (MDSC) to form a premetastatic niche that ultimately promotes liver metastases^36^. Disrupting the CXCL1/8-CXCR2 axis can suppress SMAD4-negative colorectal cancer^37^. C-X-C motif chemokine ligand 12 (CXCL12)/IRH/PBSF/SDF1 is an antimicrobial gene encodes a stromal cell-derived alpha chemokine member of the intercrine family. CXCL12/CXCR4 promotes invasion of ovarian cancer cells by suppressing ARHGAP10 expression via the VEGF/VEGFR2 signaling pathway^38^.

Collagen type I alpha 2 chain (COL1A2)/OI4/EDSCV/EDSARTH2 gene encodes the pro-alpha 2 chain of triple helix type I collagen. COL1A 2 suppresses CRC, thus it is a potential therapeutic option for CRC^39^. PI3K, Akt and p-Akt proteins are over-expressed in gastric cancer tissues, relative to adjacent normal tissues. Comparable trend has been observed for COL1A2, COL6A3 and THBS2 mRNA expression in gastric cancer tissues^40^. Aurora kinase A (AURKA)/BTAK/STK6/PPP1R47 gene product is a cell cycle-regulated kinase that participates in microtubule formation and/or stabilization of the spindle pole during chromosome segregation. In gastrointestinal cancer cell lines with activated KRAS, AURKA phosphorylates RPS6KB1, promoting proliferation, survival and growth of xenograft tumors in mice. Inhibiting AURKA slows down the growth of gastrointestinal tumors by activating KRAS^41^. Moreover, overexpression of AURKA enhances Oxaliplatin-mediated killing of colon cancer cells. Conversely, AURKA knockdown significantly weakened the chemosensitivity of colon cancer cells to Oxaliplatin^42^. Ubiquitin conjugating enzyme E2 C (UBE2C)/UBCH10/dJ447F3.2 modifies cellular abnormal or short-lived proteins destined for degradation. UBE2C not only suppresses gastric cancer colony formation, but also inhibits biosynthesis of gastric cancer DNA^43^. Over-expressed of UBE2C in rectal carcinoma modulates miR-381 expression, promoting proliferation invasion of rectal carcinoma cells but inhibits apoptosis of cells^44^. DNA topoisomerase II alpha (TOP2A)/TOP2/TP2A gene encodes DNA topoisomerase enzyme that disentangle the topological problems of dsDNA during replication and mRNA transcription. DNA topoisomerases, particularly type IIA topoisomerases, are potential therapeutic targets for numerous anticancer therapies^45^. TOP2A is an oncogene for colon cancer, and even after development, TOP2A is over-expressed in the cancer cells^46^. Aldehyde dehydrogenase 1 is a member of aldehyde dehydrogenase family encoded by HGNC (ALDH1A1)/HEL-9/PUMB1/RALDH1 gene. Herein, we found ALDH1A isoforms in multidrug resistance colorectal cancer tissues. Besides, ALDH1A is a potential marker for cancer stem cell. This revelation has opened a new frontier in to treatment of colorectal adenocarcinoma and other tumors^47^. ALDH1A1 expression is associated with poor differentiation and prognosis of primary tumors, and shorter overall survival of respective patients. Over-expression of ALDH1A1 in tumors is also associated with therapy resistance and liver metastases^48^. The beta subunit of activated cAMP protein kinase encoded by HGNC (PRKACB)/CAFD2/PKACB/PKA C-beta gene is a member of the serine/threonine protein kinase family. Downregulated expression of PRKACB is associated with shorter OS of CRC patients^49^.

Glucagon is encoded by HGNC (GCG)/GLP1/GLP2/GRPP/GLP-1 gene. The protein is composed of four distinct peptides. Glucagon is produced by the pancreas, and antagonizes the glucose-lowering action of insulin by stimulating glycogenolysis and gluconeogenesis. It is a ligand for a specific G-protein linked receptor whose signaling pathway regulates cell proliferation. Two of the four glycogen peptides are secreted in the gut of endocrine cells, and promotes nutrient absorption through various mechanisms. The fourth glycogen sub-unit is an active enteroglucagon that compares to glicentin^50^.

Overall, under-expression of ITIH2, COL1A2, TIMP1 and AURKA is associated with longer overall survival of CRC patients; whereas COL1A2 expression correlates with longer CRC free survival. In set 1, over-expression of ITIH2 was associated with poor overall survival. According to the Human Protein Atlas, ITIH2 is enriched in normal liver tissues and liver cancer tissues. However, pathological section (Human Protein Atlas) shows that the gene(ITIH2) not detected in tumor cells in colon cancer. The reason may be a insufficient number of pathological data available. Therefore, these observations are worth further in-depth study and more clinical pathological data are required to observe. In set 2, over-expression of the 3 hub genes (TIMP1, COL1A2 and AURKA) was associated with poor overall survival. In order to more accurately analyze the genes involved in colon cancer, we performed disease-free survival analysis on 10 genes. over-expression of COL1A2 was associated with poor disease-free survival of CRC patients. It further illustrates the close relationship between the gene (COL1A2) and the incidence of colon cancer. At the same time, the pathological section (Human Protein Atlas) shows that the gene is significantly expressed in colon cancer.

## Conclusion

In this study, we identified 35 DEGs between primary CRC tissues and metastatic CRC tissues. Meanwhile, 142 DEGs are identified between primary CRC tissues and normal colon tissues. Top 10 most dysregulated hub genes including AHSG, SERPINC1, FGA, F2, CP, ITIH2, APOA2, HPX, PLG and HRG predicts the development of liver metastasis in CRC patients. On the other hand, TIMP1, CXCL1, COL1A2, MMP1, AURKA, UBE2C, CXCL12, TOP2A, ALDH1A1 and PRKACB are the core genes of primary colorectal cancer. Over-expression of ITIH2 and COL1A2 is associated with better prognosis of CRC patients with liver metastasis. As such, ITIH2 might represent the potential core gene for colon cancer liver metastasis. COL1A2 behaves as a key gene in colon cancer. Nonetheless, additional studies are needed to validate our findings.

## Data Availability

The datasets used and/or analysed during the current study are available from the corresponding author on reasonable request.

## Abbreviations

BP: Biological process
CC: Cellular component
CRC: Colorectal Carcinoma
DAVID: Database for Annotation Visualization and Integrated Discovery
DEG: Differentially expressed gene
GO: Gene Ontology
GEO: Gene Expression Omnibus
KEGG: Kyoto Encyclopedia of Genes and Genomes
MF: Molecular function
PPI: Protein–protein interaction
